# Multidimensional assessment of Game Transfer Phenomena: Intrusive cognitions, perceptual distortions, hallucinations and dissociations

**DOI:** 10.3389/fpsyg.2022.896238

**Published:** 2022-08-10

**Authors:** Angelica B. Ortiz de Gortari, Åge Diseth

**Affiliations:** ^1^Faculty of Psychology, Centre for the Science of Learning and Technology, University of Bergen, Bergen, Norway; ^2^Psychology and Neuroscience of Cognition Research Unit, Faculty of Psychology, Speech Therapy and Education Sciences, University of Liège, Liège, Belgium; ^3^Faculty of Psychology, University of Bergen, Bergen, Norway

**Keywords:** Game Transfer Phenomena, involuntary phenomena, video games effects, hallucinations, perceptual distortions, dissociations, imagery

## Abstract

Game Transfer Phenomena (GTP) refers to a cluster of involuntary phenomena related to playing videogames, including sensory and cognitive intrusions, transient changes in perception and self-agency. The Game Transfer Phenomena Scale (GTPS) has been used to measure the frequency of GTP with respect to five factors. The present study aimed to validate an instrument for assessing the multiple dimensions of GTP (GTP-MDS) that helps clarify the distinction between GTP experiences. GTP were contextualized onto the spectrum of intrusive cognitions, perceptual distortions, and dissociations. The relationship between GTP, involuntary phenomena without game content (INVWG) in terms of, e.g., hallucinations and perceptual distortions, and game-biased perceptions (GBPA), as well as the positive and negative impact of GTP and level of distress were also examined. The data were collected using a survey (*N* = 1,301, male 83.4%, mean age = 28.14). Separate confirmatory factor analyses of the dimensions of “inner intrusions/misperceptions,” “outer intrusions/distortions,” and “dissociations/mix-ups” produced acceptable fit indices. The findings show that phenomena manifesting as internal experiences are more common, while those manifesting as externalized intrusions are less common. Correlations between the GTP dimensions, INVWG, and GBPA, such as the insertion of game elements in thoughts, perceptions, and dreams, supported convergent validity. The correlations between the GTP dimensions and hours played supported criterion validity. Distress was correlated with outer intrusions and dissociations/mix-ups, but not with inner intrusions. Taken together, these results support the validity and reliability of the proposed assessment of GTP constructs.

## Introduction

Involuntary or non-volitional phenomena arise spontaneously, without deliberate effort, and in an uncontrollable manner. They can manifest as thoughts or words popping into one’s mind, unintentional behaviors, or faulty sensory perceptions that generate discordance between what is perceived and what genuinely exists. Experiencing involuntary or non-volitional phenomena is relatively common after exposure to sensory stimuli or the practice of repetitive activities, such as listening to music, playing the piano (Haueisen and Knösche, 2001; Liikkanen, 2012), or playing video games (Ortiz de Gortari and Griffiths, 2016). Typical incidents include hearing music, feeling involuntary movements of the fingers, such as when playing the piano or pushing the gamepad control, and seeing images from the game.

Not much attention has been paid to involuntary phenomena related to the practice of certain activities or exposure to interactive media, including video game playing. Video game playing seemed to pose the ideal conditions for facilitating the re-experience of elements after the activity. Playing video games usually exposes the player for prolonged periods of time to rich multisensory virtual environments (e.g., images, sounds, music, and haptics) that are usually paired with in-game actions and events that elicit emotions.

A seminal interview study conducted with 42 Swedish players revealed how playing video games permeated players’ everyday lives with sensory experiences, thoughts, and automatic behaviors ([Bibr B31][Bibr B31]). In this study, Ortiz de Gortari (2010) coined the term Game Transfer Phenomena (GTP) to refer to involuntary phenomena derived from virtual interaction, particularly video games.

Game Transfer Phenomena experiences have been measured with the Game Transfer Phenomena Scale (GTPS), comprising 20 items. The scale has been validated with an international sample (Ortiz de Gortari et al., 2015), a Turkish sample (Dindar and Ortiz de Gortari, 2017), and a Polish sample (Cudo et al., 2022). The GTPS assesses GTP in terms of a five-factor structure consisting of altered visual perceptions, altered auditory perceptions, altered body perceptions, automatic mental processes, and actions/behaviors factors (cf. Ortiz de Gortari et al., 2015). Studies conducted with international samples using the original GTPS have shown a prevalence of GTP between 75 and 99% among video game players (Ortiz de Gortari and Griffiths, 2016; Dindar and Ortiz de Gortari, 2017; Ortiz de Gortari, 2017; Ortiz de Gortari and Gackenbach, 2021; Cudo et al., 2022). These studies have shown that most participants have experienced GTP more than once. However, the severity of GTP is usually low when considering both the possible forms of their manifestation and their frequency (Ortiz de Gortari et al., 2016).

The present study proposes an alternative classification of players’ experiences in a more meticulous manner, based on a consideration of the wide spectrum of involuntary phenomena, which include thoughts, imagery, misperceptions, perceptual distortions, hallucinations, and dissociations. This has been accomplished by validating a new scale, the Game Transfer Phenomena Multidimensional Scale (GTP-MDS), which attempts to discriminate between the types of GTP and thus facilitates the identification of its underlying physiological, cognitive, and etiological mechanisms. This study also investigates the positive/negative impacts and experiences of distress associated with GTP; even though most players tend to appraise GTP as positive (Ortiz de Gortari and Griffiths, 2016). The potential risks associated with GTP have been argued to depend upon how players appraise their GTP experiences and the contexts in which GTP manifest (Ortiz de Gortari, 2019).

### Game Transfer Phenomena and related fields of research into media effects

Wesener (2004); Fritz (2005), and Witting (2007) investigated the transfer of experiences from video games from a learning perspective. Fritz identified ten different types of transfers such as problem-solving, emotional, and associative when establishing a connection between different stimuli. Bigl (2013) found that in over a thousand participants, most (87%) reported some of the transfer of experiences proposed by Fritz. The most common were transfers of dreams and knowledge.

Other researchers have examined the transfer of experiences focusing on game-biased perceptions or associations via the Game-Bias Perceptions and Associations Scale (GBPA). The GBPA was developed by Poels et al. (2014) and assesses automatic associations between physical stimuli, such as objects and sounds that trigger memories from a game. While the GBPA does not distinguish between voluntary and involuntary phenomena as the GTP framework (Ortiz de Gortari, 2016, 2019), it does account for integrating video game content used in everyday jargon and nightly dreams with video game content.

The cultivation theory was proposed by Gerbner and Gross (1976) and originated from television studies in the 1970s. This theory posits that media shapes the perception of social reality, beliefs and attitudes. Studies focusing on video games have found that the cultivation effects of playing games are limited and specific to the content of the game, rather than affecting the broader perception or expectations the player has of the real world (Lee et al., 2010; Chong et al., 2012).

The Proteus effect, coined by Yee and Bailenson (2007), explores the embodiment and identification with avatars and has shown how people conform their behaviors and attitudes with their avatars’ characteristics. This theory has been extensively investigated in experimental settings and highly immersive environments. Changes in behaviors have been observed, for example, how people choose dating partners, negotiate with more aggressiveness, behave as a child or as an older person, or show prosocial or antisocial behaviors (Ratan et al., 2020).

The video-terminal dissociative trance (VTDT) proposed by Schimmenti and Caretti (2017) argues that the interaction with computers and related applications can result in “significant disturbances in the states of consciousness, identity, and memory, the dilution of self-awareness and self-integrity, and the replacement of the customary sense of personal identity by a new virtual identity” (Schimmenti and Caretti, 2017 p., 64).

### Nature and characteristics of Game Transfer Phenomena

Game Transfer Phenomena experiences are situated on the continuum between common everyday involuntary phenomena (e.g., inner speech, music stuck in one’s head, mental visualizations) and those experiences classified as symptoms of disorders, in which the intrusions or distortions of reality are recurrent and provoke distress and dysfunction for over a long period of time (Ortiz de Gortari, 2019).

Typical forms of GTP reported in previous studies include hearing a game’s music, which resembles the typical music imagery one experiences after listening to music. Another common form of GTP is seeing game images when blinking or closing one’s eyes (Ortiz de Gortari and Griffiths, 2016; Ortiz de Gortari et al., 2017). The manifestations of GTP include inner (e.g., hearing sounds inside the head) or outer phenomena (e.g., seeing images overlaying physical objects; Ortiz de Gortari and Griffiths, 2014b,^2016^). The duration tends to be short-lived (i.e., seconds and minutes), and they are more likely to occur while an individual is engaged in day-to-day activities triggered by game-related cues than while trying to fall asleep or when waking up (Ortiz de Gortari and Griffiths, 2016). In most cases, players are aware that what they see or hear is not real; therefore, the sense of reality remains intact (Turner, 2014).

According to a multimodal and holistic theoretical approach to GTP (Ortiz de Gortari, 2016, 2019), it is important to differentiate their forms of manifestation and characteristics to fully understand the nature of these phenomena (i.e., the physiological, perceptual, and cognitive underpinnings of GTP), as well as their impact (i.e., in areas of daily functioning such work, relationships and level of distress caused). The following section provides an overview of the aspects taken into consideration in the selection of items for the proposed MSD-GTP. These aspects include (i) differentiation of involuntary from voluntary phenomena, (ii) identification of the spatial location of GTP, (iii) identification of whether environmental stimuli act as triggers of GTP, and (iv) differentiation of the complexity of GTP.

#### Differentiation of involuntary from voluntary phenomena

The items proposed for assessment by the new scale make it clear that the players’ experiences occurred involuntarily, without premeditation, and without any or very restricted control on the part of the players. This contrasts with volitional/voluntary phenomena denoted by players intentionally integrating video game content into their behaviors, which can also involve premeditation. Some examples of voluntary phenomena include reenacting video game content for fun, using slang or game expressions to communicate with friends, and integrating video games into schoolwork (Ortiz de Gortari and Griffiths, 2014c).

#### Identification of the spatial location of Game Transfer Phenomena experiences

Distinguishing the location of sensory intrusions with game content is important for various reasons. First, identification of the inner or outer spatial location of GTP may contribute to understand the nature of such experiences. On the one hand, some inner phenomena, such as mental visualizations and inner speech, appear to be better explained by memory mechanisms. On the other hand, continuing to see images in the back of the eyelids or the persistent recurrence of a visual image after the stimulus has been removed resemble palinopsia i.e., the perseveration and recurrence of images (Takayama et al., 2019) and involve neural adaptative mechanisms. In the case of GTP, such experiences are most likely due to the exposure to visual stimuli for a prolonged period of time when playing a game (Ortiz de Gortari and Griffiths, 2017; Ortiz de Gortari et al., 2017). These perceptual intrusions also resemble afterimages as the side effects of using hallucinogenic drugs, referred to as hallucinogen persisting perception disorder (HPPD), when the intrusions become recurrent and provoke dysfunction (Kilpatrick and Ermentrout, 2012). When images arise in the transitional state between being awake and falling asleep, they are considered hypnagogic hallucinations (Mavromatis, 2010), although others have referred to images that occur during the daytime as parahypnagogia (Gurstelle and de Oliveira, 2004).

Second, evidence suggests that, depending on the perceived location of the GTP (outer or inner), the impact of the experience can vary (Ortiz de Gortari and Griffiths, 2014a,b, 2016). Outer or external GTP can facilitate (i) the interpretation of the intrusion as alien, rather than self-generated, causing confusion as the experiences tend to be considered bizarre; (ii) the elicitation of illogical thoughts with expectations that events from the game will occur in real life, which, depending on the game content, can be aversive; and (iii) the triggering of automatic behaviors or impulsive actions toward the intrusions that are heard or seen, which in certain circumstances can represent a risk (e.g., seeing images when driving). Inner or internal GTP, such as continuing to hear music in the head or visualizing images in the back of the eyelids, has been reported to lead to sleep deprivation (Ortiz de Gortari and Griffiths, 2014a).

#### Identifying the involvement of environmental stimuli as triggers of Game Transfer Phenomena experiences

Game Transfer Phenomena can occur in different circumstances. Some types of GTP happen when the players are exposed to limited or no external stimuli, such as in darkness as one is trying to fall asleep (Ortiz de Gortari and Griffiths, 2016). Re-experiencing game content under hypnagogic states has been induced experimentally (Wamsley et al., 2010; Kusse et al., 2012), and even amnesic patients have reported it (Stickgold et al., 2000), suggesting that re-experiencing game content under these circumstances does not involve declarative memory systems. GTP can also occur when engaging in everyday activities without the involvement of environmental triggers. An example of a GTP without a trigger is when images from the game continue to appear immediately after playing or when looking away from the screen (e.g., seeing images floating on a wall). These experiences appear to be explained by neuroadaptive mechanisms due to overstimulation or sensitivity of the eyes’ photoreceptors (Ortiz de Gortari and Griffiths, 2014a; Ortiz de Gortari, 2017). Another commonly reported manifestation of GTP are those that are triggered by external stimuli associated with the game (Ortiz de Gortari and Griffiths, 2016). These types of GTP experiences suggest the involvement of implicit learning (Voss et al., 2010) and the influence of expectations, beliefs, and emotions on perceptions (Powers et al., 2016). Examples range from seeing power bars above peoples’ heads, or seeing images from the game Guitar Hero after hearing the word “guitar” (Ortiz de Gortari and Griffiths, 2014a). Table 1 shows examples of the items developed with consideration of environmental triggers.

**TABLE 1:** Examples of the adaption of the items of the original GTP Scale to this new revised and extended version.

GTPS items	New items in the GTP-MDS	
Original item	Inner phenomena without implicit trigger	Outer phenomena with implicit trigger
I have heard the music from a game when I was not playing I have heard sound effects from a game when I was not playing.	I have heard music or sounds from a video game inside my head. Example: heard melodies, steps, or gunshots from a video game in my head.	I have heard music or sounds from a video game outside my head as if coming from somewhere or from nowhere. Example: heard sounds from a video game coming from the console even though it was shut down; heard video game music in a room even though nothing was there.
I have seen video game images with my eyes open when I am not playing (e.g., seeing health bars above peoples’ heads or maps in the corner of the eye).	I have seen video game elements with my eyes open, without seeing, hearing, or doing something else associated with a game. Example: seen a video game image floating on the wall.	I have seen video game elements with my eyes open while seeing, hearing, or doing something associated with a game. Example: seen a video game conversation menu while in a dialog; seen health bars above people’s heads.

#### Differentiating the complexity of Game Transfer Phenomena experiences

Some forms of GTP manifest as simple thoughts, misperceptions (e.g., thoughts about the game or associations between physical stimuli and game content, such as perceiving game elements in ambiguous objects, akin to pareidolia), or as sensory residuals, such as seeing images from the game every time one closes their eyes (Ortiz de Gortari and Griffiths, 2014a). In other cases, a GTP experience may manifest as a more complex phenomenon involving disconnecting from one’s ongoing activity, thoughts, feelings, or sense of reality or identity in an experience more related to dissociative phenomena. For instance, experiencing sensations of unreality and source monitoring errors between virtual and real-life events appears to involve failures of stimulus discrimination, like flashbacks in PTSD when encountering stimuli associated with a traumatic event (Ortiz de Gortari and Griffiths, 2014c,^2017^) that lead to active avoidance and hyperarousal (Felmingham et al., 2002). Complex forms of manifestations of GTP can also lead to involuntary actions due to poor impulse control or poor control of body movements (due to a lack of coordination or postural disequilibrium), which can be explained by neural adaptations enhanced by prolonged immersion in the virtual world (Champney et al., 2007).

## Aim and hypotheses

The main aim of this study was to validate a new instrument for measuring GTP dimensions by investigating its factor structure. A secondary aim was to determine how the GTP dimensions were related to general involuntary phenomena [i.e., involuntary phenomena not related to playing video games or video game content; involuntary phenomena without game content (INVWG)], game biases (GBPA), and playing time to test the convergent and criterion validity of the GTP dimensions. Finally, this study also investigated the impact of experiencing GTP (positive impact, negative impact, and distress) for the different GTP dimensions.

We hypothesized the following regarding the proposed new instrument for assessing GTP:

H1. Dividing the items into the dimensions “inner intrusions” and “outer intrusions,” which contrast the perceived location of the GTP experiences, and “dissociations/mix-ups,” which focus on experiences that involve self-agency, body sensations, and mix-ups between the game and reality, will provide a good fit and reliability.

Game Transfer Phenomena are understood as involuntary phenomena, and it is expected that individuals susceptible to involuntary phenomena in other contexts (e.g., hallucinations or imagery) will also be susceptible to GTP; therefore, we hypothesized the following:

H2. Susceptibility to general intrusions and game-biased perceptions will be associated with all GTP dimensions.

Lastly, GTP manifesting as outer intrusions or involving dissociations have been associated with reports of distress and risky behaviors (Ortiz de Gortari et al., 2011; Ortiz de Gortari and Griffiths, 2014c). Hence, the following was expected:

H3. Experiencing GTP in the outer intrusion dimension or the dissociations/mix-ups dimension will be associated with distress and a negative impact.

## Materials and methods

### Participants

The participants (*N* = 1,301, mean age = 28.14, range 18–61 years) were video game players recruited via online outlets. The majority were male (83.4%), did not have a clinical or neurological diagnosis (78.7%), and had never used drugs (64.3%). Most of the participants were employed (50.3%) or students (30.9%). The most common places of residence were Mexico (28.3%), Sweden (19.26%), United States (15.8%), United Kingdom (4.9%), Norway (3.38%), Canada (2.6%), and Germany (2.6%). The majority played video games for more than 10 years (87.7%). More than half (65.4%) played five or more days a week and played on average 3.86 h a day in average sessions of 2.89 h. The average hours per week were 20.65 (Sd = 16.771). Almost half, 44.4%, played 2–3 h per day, and another 28.7% played 4–5 h per day. A total of 3.5% played esports professionally. The video game genres played by more than half of the sample were Adventure (66.4%), Role-playing (63.3%), and FPS (62%).

### Material

A series of questions were created to define the participant profile in terms of demographics, playing habits, and clinical profile. Specifically, the questions included:

#### Demographic

These questions included gender, age, country, and occupation.

#### Playing habits

These questions assessed playing time, i.e., days per week, playing time per day and session length, years of experience playing, and most frequently played genres.

#### Impact of Game Transfer Phenomena

Participants who reported having experienced at least one instance of GTP could rank the distress, positive or negative impact associated with their GTP in some area of daily functioning (e.g., work, relationships) via three different ranking scales from 1 not at all through distressing/positive/negative to 5 very distressing/positive/negative.

**Involuntary phenomena without game content (INVWG)** items from various scales on unusual phenomena, hallucinations, and sleep disorders were selected to test the convergence with the GTP items. Seven items which include measures of seeing, hearing something, tactile sensations, and changes in visual and auditory perception of things, persons, colors, or sounds, were selected from the **Cardiff Anomalous Perceptions Scale (CAPS)** (Bell et al., 2006), which measures perceptual anomalies using a dichotomy answers (yes or no). E.g., “do you ever see shapes, lights, or colors even though there is nothing really there?” “Do you ever notice that sounds are much louder than they normally would be?” Cronbach’s Alpha was 0.808. Another five items, which assess vivid imagery and memories, confusion between dreams and genuine events, detachment of body, and derealization, were borrowed from the Dissociative Experiences Scale II. In the DES-II, respondents choose a percentage (e.g., 10%) indicating how often they experience different dissociative symptoms (Carlson and Putnam, 1993) in terms of e.g., “some people have the experience of sometimes remembering a past event so vividly that they feel as if they were reliving that event,” “some people have the experience of feeling that their body does not seem to belong to them.” Cronbach’s Alpha was 0.828. Lastly, three items were included from the **Iowa Sleep Experiences Survey (ISES),** which measures general sleep disorders phenomena related to body movements such as twitches and jerks, and vivid images as a lying-in bed or fall asleep, e.g., “My legs twitch or tremble as I lie in bed,” “I experience intense, dreamlike images as I begin to fall asleep.” Cronbach’s Alpha was 0.690.

**Post-play video game phenomena triggered by associations** were assessed by means of the **Game-biases perceptions and associations (GBPA)** (Poels et al., 2014). This instrument was validated on video game players of massive multiplayer online role-playing games (MMORPG) and proposed five dimensions: physical objects, sound and music, vocabulary and expressions, daydreams, and nightly dreams. A total of 12 of 22 items were used from the domains: (i) memories from the game triggered by physical objects, (ii) daydreams, and (iii) memories from the game triggered by sound and music. Cronbach’s Alpha for these items was 0.859.

### The scale development

In order to measure GTP, a total of 46 items were created using a five-point scale of frequency (never, once, a few times, many times, all the time). Involuntary phenomena reported by video game players via qualitative studies (Ortiz de Gortari and Griffiths, 2014a,b,c) were included together with the 20 items of the original GTP scale (Ortiz de Gortari et al., 2015), which were rephrased to assess broader characteristics of GTP. Table 1 shows examples of how items from the original GTP scale (Ortiz de Gortari et al., 2015) were transformed.

The new items assess GTP in various sensory channels (i.e., visual, auditory, kinesthetic, and tactile), thoughts, and actions/behaviors. The items range in diversity from phenomena common among the general population, such as imagery (Giambra, 2000; Liikkanen, 2012), to more unusual phenomena commonly associated with clinical and neurological disorders, trauma, or substance intoxication, such as dissociations and hallucinations (Ohayon, 2000; Preti et al., 2014). The items created were classified as follows:

• **Inner intrusions/misperceptions.** This factor comprised inner/endogenous phenomena, which included intrusive thoughts (e.g., “focused attention on objects related to the video game”), misperceptions/mental imagery (e.g., “seeing images with closed eyes”), recognizing patterns in ambiguous stimuli (i.e., pareidolia), and inner auditory experiences (e.g., “words/phrases/music repeated/stuck in the head”).
• **Outer intrusions/distortions.** This factor comprised visual and auditory intrusions that are perceived as externalized, such as images overlaying a physical context (e.g., seeing power bars above peoples’ heads), sounds coming from the console even though is turned off, and perceiving objects, sounds, or environments as distorted based on the video game characteristics (e.g., seeing environments tinted with the color scheme of the game).
• **Dissociations and mix-ups.** This factor comprised items that assessed aspects related to body sensations, thoughts, and behaviors that involved source monitoring errors, slips of actions, and dissociations. Specifically, such aspects consist of confusions and mix-ups between virtual and physical stimuli, perceptual changes, body sensations related to virtual embodiment and presence in the virtual world (probably because of neural adaptations and repetitive in-game behaviors/actions), impulses to act as in the game, and actions enacting the game in the physical world. Examples include the sensation of still being in the game environment, feeling changes in one’s body, feeling an urge or acting upon a game-related stimulus, involuntary movement of the limbs, or verbal outbursts.

Table 2 shows the classification of each item according to the perceived location, type of phenomena, and sensory modality. The instructions indicated that respondents were only to report GTP that had been experienced when not under the influence of a substance: “Please only report experiences you had when NOT been under the effect of some psychoactive substance (e.g., alcohol, cannabis, ecstasy, etc.).”

**TABLE 2:** Classification of GTP according to perceived location, type of phenomena and the sensory modality.

Perceived location	Thoughts/impulses	Visual	Auditory	Corporeal/actions	Kinesthetic	Chronoception	Multimodal
Inner intrusions/ misperceptions	-Recurrent thoughts -Selective attention toward game-related stimuli -Mind popping	-Afterimages -Mind visualizations -Visual misperceptions/ interpretations of ambiguous stimuli	-Auditory imagery -Auditory mind-popping -Inner-speech -Gedankenlautwerden* -Auditory misperceptions				
Outer intrusions/ distortions		-Visual hallucinations with triggered/without triggered -Visual hallucinations that lead to action -Distortion of external stimuli	-Music, sound, voices externalized -Distortion of external stimuli				
Dissociations and mix-ups	-Urge of impulses toward game related stimuli -False expectations -Memory mix-ups -Action mix-ups -Perseverative procedural mental processes			-Tactile sensation -Distortion of body -Body detachment -Body attachment to video game elements -Derealization -Depersonalization -actions (verbal outburst, change of behavior, and responses toward game-related stimuli.)	-Sensation of body movement -Involuntary movements of limbs -Sensation of movements of limbs	-Distortion of velocity and time	-Absent/catatonic-like

*Consider a verbal hallucination as thought-echo, thought echoing, thoughts-out-loud or audible thinking (Spitzer, 1988).

The item assessment included the location and involvement of environmental triggers, as well as an overall scale assessing the impact of GTP (i.e., negative/positive impact on daily functioning and degree of distress).

#### Evaluation of the Game Transfer Phenomena-multiple dimensional scale items

A panel of experts and video game players was consulted, and the evaluation consisted of the following:

##### Evaluation by a panel of experts

In the first stage, the items were evaluated by a panel of ten experts from various disciplines, including cognitive neuroscience, clinical psychology, psychiatry, cognitive psychology, and more specific areas, such as behavioral addiction, involuntary memories, and visual stress. The evaluation was conducted in three steps: (i) completing the scale to become familiar with the items, (ii) providing general feedback on the scale, in which the reviewers rated the structure of the scale; and (iii) providing feedback on the content of the items. The panel of experts thus evaluated the structure of the scale and the individual items.

• **Structure of the scale.** This part included evaluations of the scale in terms of (i) instructions, (ii) the frequency scale used, (iii) the language used in the items, (iv) the design of the scale (e.g., organization and number of items), and (v) the labeling of the GTP dimensions. With respect to the evaluation criteria, aspects that were rated as poor (5) or fair (4) by most of the reviewers would be reviewed and modified.
• **Items.** The items were evaluated using dichotomy questions in terms of (i) coverage of a wide variety of experiences in the different domains, (ii) clear differentiation or distinctiveness between items, (iii) whether the items assessed what was intended to be measured, (iv) clarity of the examples that accompanied each item, and (v) discrepancies found between items and examples.

##### Evaluation by video game players or users

Five video game players were invited to evaluate the items created for the new assessment of GTP. The aim of the evaluation was to verify whether the items were clear enough, identify difficulties answering the items, and determine what thoughts or feelings were prompted (if any) by answering the questionnaire. After the players responded to the GTP items, they rated the degree of clarity of the items and the examples using a scale of five points (i.e., extremely clear, very clear, somewhat clear, slightly clear, and not at all clear). Moreover, they were asked to comment on the thoughts and feelings that might arise when answering the questionnaire.

### Procedure

The items to assess GTP were modified based on the feedback of the expert panel and the video game players. The next step consisted of conducting a survey to test the new items. Participants were recruited via online outlets (e.g., online forums, Facebook, university notice boards, and redirected emails). The study protocol and procedures were approved by the ethical committee of the university.

### Statistical analyses

A series of confirmatory factor analyses (CFA) were conducted to assess the factor structure of the proposed new items. SPSS AMOS Graphics software was used to perform the analyses. The comparative fit indices (CFI) and the root mean square error of approximation (RMSEA) were utilized as the main indicators of model fit. Here is an overview of the analyses performed:

• Factor structures for each dimension of the GTP-MDS was analyzed by means of CFA.
• Descriptive statistics for all variables (dimensions and subscales of the GTP-MDS) were provided.

Furthermore, the GTP-MDS dimensions were correlated with the following variables as means of investigating criterium and convergent validity:

• Amount of video game playing (days, hours, and session length).
• Cardiff Anomalous Perceptions Scale, DES, ISES, and GBPA.
• Finally, Pearson correlations were performed to examine associations between the impact of GTP and the different dimensions of GTP.

## Results

### Confirmatory factor analyses for the dimensions of Game Transfer Phenomena

Three separate CFA were performed, in which each of the GTP dimensions (inner intrusions/misperceptions, outer intrusions/distortions, and dissociations and mix-ups) accounted for the variance in particular subscales with a final total of 38 items (a copy of the GTP-MDS and instructions can be found at gametransferphenomena.com).

### Confirmatory factor analyses for inner intrusions/misperceptions

This dimension comprises a broad range of inner/endogenous phenomena manifesting as intrusive thoughts, visual and auditory mental imagery internally generated or triggered by environmental stimuli (e.g., keep hearing music, sound or speech in the head, identifying game objects in ambiguous patterns). A total of 12 items were accounted for by a three-factor structure with acceptable fit indexes (Chisq = 192.99, df = 50, Chisq/df = 3.86, CFI = 0.97, RMSEA = 0.047), according to guidelines by Kenny et al. (2015). Factor 1 comprises intrusive thoughts that are typically elicited by game-related cues. Factor 2 comprises mental imagery, visualizations behind the eyelids, and misperceptions. Factor 3 comprises endogenous auditory phenomena that include hearing sounds, music, voices, and internal monolog (see Table 3). The covariances between the latent factors were 0.83 (F1–F2), 0.88 (F2–F3), and 0.79 (F1–F3).

**TABLE 3:** Standardized factor loadings for the inner intrusions/misperceptions dimension.

	F1	F2	F3
**Intrusive thoughts**			
Intrusive thoughts	0.60		
Focused attention to objects related to a VG	0.69		
Thought of using/wanting to use a VG element or ability	0.66		
**Misperceptions/mental imagery**			
Images with closed eyes		0.55	
Mind visualizations		0.68	
Visual misperceptions		0.50	
Pareidolia		0.64	
**Inner auditory**			
Music/sounds in the head			0.64
Words/phrases repeated/stuck in the head			0.72
Word/phrase manifest as a single instance			0.66
Inner speech preserving game characteristics			0.57
Talked to oneself in the head using style/vocabulary from a VG			0.62

F1 = Intrusive thoughts, F2 = misperceptions/mental imagery, F3 = Inner auditory.

### Confirmatory factor analyses for outer intrusions/distortions

This dimension measures external or outer visual and auditory hallucinations and visual and auditory perceptual distortions (e.g., saw images with open eyes with/without trigger, heard music or sounds outside the head). In order to produce an appropriate factor solution, four of the 11 items were deleted, resulting in a seven-items in a one-factor structure with acceptable fit indexes (Chisq = 43.919, df = 12, Chisq/df = 3.66, CFI = 0.98, RMSEA = 0.045) (see Table 4).

**TABLE 4:** Standardized factor loading for items in the outer phenomena dimension.

	F1
Saw images with open eyes with a trigger	0.61
Saw images with open eyes without a trigger	0.55
Heard music or sounds outside the head	0.55
Heard a voice outside the head	0.65
Saw images and interacted with them	0.50
Heard music, sounds or voices distorted or changed	0.64
Saw objects or environments as having distorted colors	0.54

F1 = hallucinations and perceptual distortions.

### Confirmatory factor analyses for dissociations and mix-ups

This dimension comprises sensations, thoughts, and behaviors specifically reflecting dissociations and involving somatosensory perceptions (e.g., sensations of whole-body movement, game elements belonging to the body), involuntary behaviors (e.g., unintentionally sang, shouted, or said something), and mix-ups (e.g., for moments believed that video game events, characters or elements happened or were real). A total of 19 items were accounted for by a three-factor structure with acceptable fit indexes (Chisq = 682.96, df = 146, Chisq/df = 4.678, CFI = 0.93, RMSEA = 0.053). Factor 1 comprises dissociations such as derealization and depersonalization and corporeal, time, and velocity altered perceptions that typically accompany dissociative states observed in neurological conditions such as seizures but also neural adaptations due to virtual immersion. Factor 2 comprises involuntary behaviors when responding to external stimuli as they were video game elements due to slips of action involving body movements or actions. Factor 3 comprises cognitive mix-ups between the physical and the virtual world stimuli, responses to external stimuli that resemble or remind one of video game elements due to cognitive slips, memory errors, and stereotypical or perseverative mental states (see Table 5). The covariances between the latent factors were 0.87 (F1–F2), 0.92 (F2–F3), and 0.85 (F1–F3).

**TABLE 5:** Standardized factor loadings for items in the dissociations/mix-ups dimension.

	F1	F2	F3
**Dissociations/corporeal adaptations**			
Distortions of velocity/time	0.62		
Sensation of whole-body movement	0.64		
Tactile sensation	0.57		
Body distortion according to the characteristics/features of characters	0.59		
Game elements belonging to body	0.64		
Sensations of still being in the game environment	0.59		
Sensation of being a game character	0.62		
Body detachment or third person view	0.54		
Catatonic state	0.52		
**Behavior mix-ups**			
Unintentionally moved limbs triggered by a game-related cue		0.65	
Sensations of limbs moving, contracting, jerking, or twitching		0.61	
Unintentionally walked, made gestures, or changed body posture		0.67	
Unintentionally sang, shouted, or said something from a game		0.57	
Acted/behaved differently towards an object, place, or situation		0.67	
**Cognitive mix-ups**			
Felt the urge to do something but did not act on it			0.60
Expected or assumed that something from the VG would happen			0.66
Thought that events, characters, or elements really happened or were real			0.57
Wanted to use a VG control/element to perform an action			0.62
Stereotypical mental replays of the game			0.67

F1 = dissociations/corporeal adaptations, F2 = action mix-ups, and F3 = cognitive mix-ups.

### Alternative factor structures

Due to the strong correlations between the factors (see Table 7), an alternative factor structure was tested in which a higher-order factor accounted for all three CFA’s. This factor structure produced parameter values above 1, indicating multicollinearity (Deegan, 1978). Hence, this solution was not admissible.

### Descriptive statistics

Descriptive statistics (Table 6) showed mean values between 1.374 and 2.321. The GTP dimensions showed good internal consistency with Cronbach’s alpha coefficients from 0.745 to 0.904.

**TABLE 6:** Descriptive statics for each of the GTP dimensions.

	Mini	Max	Mean	Sd	Skew.	Std.	Kurt.	Std.	Cronbach’s
	Stat	Stat	Stat	Stat	Stat	Error	Stat	Error	alpha
**Inner intrusions**	1.00	4.56	2.190	0.759	0.392	0.068	−0.478	0.136	0.790
Intrusive thoughts	1.00	5.00	2.321	0.986	0.270	0.068	−0.851	0.136	0.678
Misperceptions/mental imagery	1.00	4.75	2.112	0.815	0.422	0.068	−0.823	0.136	0.665
Inner auditory	1.00	5.00	2.137	0.853	0.622	0.068	−0.845	0.136	0.784
**Outer intrusions (hallucinations and perceptual distortions)**	1.00	4.14	1.317	0.495	2.260	0.068	5.816	0.136	0.779
**Dissociations/mix-ups**	1.00	4.11	1.452	0.534	1.753	0.068	3.375	0.136	0.904
Dissociations/corporeal adaptations	1.00	4.00	1.374	0.508	1.929	0.068	4.098	0.136	0.822
Behavior mix-ups	1.00	4.40	1.415	0.610	1.509	0.068	1.639	0.136	0.766
Cognitive mix-ups	1.00	4.11	1.567	0.683	1.404	0.068	0.558	0.136	0.745

The bold text indicates each of the dimensions of GTP.

**TABLE 7:** Correlations between the dimensions of GTP.

		1	2	3	4	5	6	7	8	9
1	**Inner intrusions**	1.00								
2	Intrusive thoughts	0.86**	1.00							
3	Misperceptions	0.85**	0.58**	1.00						
4	Inner auditory	0.86**	0.59**	0.65**	1.00					
5	**Outer intrusions^1^**	0.57**	0.38**	0.52**	0.58**	1.00				
6	**Dissociations/mix-ups**	0.71**	0.59**	0.60**	0.65**	0.69**	1.00			
7	Dissociations	0.62**	0.47**	0.54**	0.58**	0.69**	0.87**	1.00		
8	Behavior mixed-up	0.58**	0.47**	0.48**	0.55**	0.60**	0.89**	0.68**	1.00	
9	Cognitive mixed-up	0.69**	0.60**	0.57**	0.60**	0.56**	0.90**	0.68**	0.69**	1.00

^1^A single factor, which includes hallucinations and perceptual distortions.

**Correlation is significant at the 0.01 level (2-tailed).

There were somewhat high skewness and kurtosis values for outer intrusions, dissociations/mix-ups, and dissociations/corporeal adaptations. However, because the standard errors for skewness and kurtosis decrease in large samples, the skewness and kurtosis values may be large even when there are minimal deviances from normality. In these cases, high skewness and kurtosis values do not deviate enough from normality to make a difference (Tabachnick et al., 2007). In addition, there are no official cut-off criteria for skewness and kurtosis, and some authors argue that skewness between −2 and 2, and kurtosis between −7 and 7 are acceptable (Curran et al., 1996; Byrne, 2010).

In order to examine the prevalence of GTP, we excluded those participants who responded “never” on the GTP-MDS items. Those who answered any of the other alternatives (“once,” “a few times,” “many times,” or “all the time”) were considered participants who experienced GTP. The results of this analysis showed that 95.2% experienced some form of GTP at least once during the last 12 months. Analyses focusing on each of the dimensions showed that most participants reported inner intrusions (94.7%), more than half reported dissociations/mix-ups (76.5%), and almost half reported outer phenomena (49.3%).

Finally, we examined the distribution of the GTP-MDS items according to the floor effects or ceiling effects (i.e., the proportion of participants who obtained the lowest possible and the highest possible score). Floor or ceiling effects are considered to be present if more than 15% of the responses have the lowest or the highest possible score (Terwee et al., 2007). For the three scales together (38 items), the lowest possible score was 38, and the highest possible was 190. Only 4.8% had the lowest possible score, while none had the highest possible score. Hence, we can conclude that there were no substantial floor or ceiling effects.

The standard error (SE) of the mean was calculated to assess the response stability of the participants. For the inner intrusions/misperceptions, it was 0.051, for the outer intrusions/distortions, it was 0.02, and for the dissociations, involuntary behaviors, and mix-ups, it was 0.06. A SE mean equal to two or less is considered good precision (Wuang et al., 2012). Hence, the response stability was good.

The Pearson correlations of the seven subscales in the dimensions of GTP ranged from 0.541 to 0.897 (see Table 7).

### Criterion-validity

In order to find further support for the validity of the GTP-MDS, we investigated how each dimension correlated with indicators of actual gaming behavior. This behavior was operationalized as the number of days playing per week, number of hours of gaming per day, and duration of gaming (session length). The average hours playing per day was 3.86 h (Sd = 2.459), and the session length was 2.89 h (Sd = 1.757). All three dimensions were significantly correlated with hours of play per day (see Table 8). Only the outer intrusion scale was correlated with session length (*r* = 0.07, *p* < 0.05). None of the dimensions correlated with days of playing per week (see Table 8).

**TABLE 8:** Correlation between GTP dimensions, playing habits (Criterium validity) and general involuntary phenomena, and associative phenomena with game content (Convergent validity).

	Days played p/week	Hours played per/day	Session length	CAPS	DES	ISES	GBPA
Inner intrusions	0.038	0.072*	0.016	0.491**	0.407**	0.450**	0.636**
Outer intrusions	−0.027	0.145**	0.073**	0.519**	0.400**	0.312**	0.306**
Dissociations/mix-ups	−0.019	0.130**	0.043	0.581**	0.520**	0.407**	0.503**

CAPS, Cardiff Anomalous Perceptions Scale; DES, Dissociative Experiences Scale; ISES, Iowa Sleep Experiences Survey; GBPA, Game-biases perceptions and associations.

**Correlation is significant at the 0.01 level (2-tailed).

*Correlation is significant at the 0.05 level (2-tailed).

### Convergent validity

The convergent validity of the scale was tested by correlating the general involuntary phenomena (INVWG) and game-biased perceptions and associations (GBPA) with the GTP dimensions.

The Pearson correlations showed moderate to high correlations between the GTP dimensions and factors and general involuntary phenomena. The correlations ranged from 0.311 to 0.583. The correlations between GBPA and GTP dimensions? ranged from 0.314 to 0.629. The highest correlation computed was between inner intrusions and the GBPA (0.629), and the lowest was between outer phenomena and the GBPA (see Table 8).

### Impact of Game Transfer Phenomena

Participants who answered affirmative to at least one GTP item were further asked to indicate the extent to which the experience of GTP had positive and/or negative impact on their daily functioning (e.g., relationships, work) and the degree of distress associated with their GTP. Overall, most participants reported a positive impact of GTP (86.9%), while only 36.3% reported some negative impact. Moreover, only 26.8% reported distress due to GTP. A correlation analysis was performed to investigate how each GTP dimension was related to distress, positive impact, and negative impact. Positive and negative impact showed lower correlations in all the GTP dimensions. The only difference observed was that inner intrusions were not correlated with distress, while outer intrusions and dissociations/mix-ups showed lower correlations (see Table 9).

**TABLE 9:** Correlation between GTP dimensions and consequences of GTP.

	Distress	Positive impact	Negative impact
Inner intrusions	0.006	0.079**	0.131**
Outer intrusions	0.179**	0.111**	0.157**
Dissociations/mix-ups	0.132**	0.137**	0.158**

**Correlation is significant at the 0.01 level (2-tailed).

## Discussion

The present study investigated a wide variety of GTP along the spectrum of imagery, illusions, perceptual distortions, hallucinations, and dissociations. The validation of the new instrument for measuring GTP consisted of (i) performing factor analyses for the three dimensions of GTP, (ii) examining how the three dimensions were related to general involuntary phenomena, game biases, and playing time to test the convergent and criterion validity of the GTP dimensions, and, lastly, (iii) determining how each GTP dimension was related to positive impact, negative impact, and distress.

## Factor analysis of the Game Transfer Phenomena

Three separate factor analyses (38 items) of the GTP-MDS showed that this instrument could effectively measure the dimensions of “inner intrusions/misperceptions,” “outer intrusions/distortions,” and “dissociations/mix-ups,” which produced an acceptable model fit and good reliability. The “inner intrusion” dimension was accounted for by the subscales of intrusive thoughts, misperceptions/mental imagery, and inner auditory. The “outer intrusions/distortions” dimension was accounted for by a single factor, labeled as hallucinations/perceptual distortions. Lastly, the “dissociations/mix-up” dimension was accounted for by dissociations/corporeal adaptations, behavior mix-ups, and cognitive mix-ups.

The “inner intrusions/misperceptions” dimension was the most prevalent, showing the highest mean value. This was expected, as this dimension measured mental imagery, inner auditory intrusion, and intrusive thoughts, which are phenomena very common among the general population (Hyman et al., 2012; Zavagnin et al., 2014). In contrast, the “outer intrusions/distortions” dimension had the lowest mean value. This finding may be explained by the fact that this dimension includes hallucinations (e.g., seeing images overlaying objects or hearing sounds coming from the environment), which are less common in non-clinical populations (Ohayon, 2000; Preti et al., 2014).

### Criterion and convergent validity

To further assess the psychometric properties of the GTP dimensions, the second aim was to test the criterion validity and convergence validity. Playing time as an indication of the exposure to game content was used as the measure of criterion-related validity. Increases in playing time have been found to be associated with GTP (Ortiz de Gortari et al., 2015, 2016; Dindar and Ortiz de Gortari, 2017), but discrepancies have also been found between hours played a week and playing session length. Some studies have even found that the hours played per day were not associated with GTP (Ortiz de Gortari and Gackenbach, 2021). The validation of the original GTPS used session length for criterion-related validity (Ortiz de Gortari et al., 2015; Dindar and Ortiz de Gortari, 2017). In the present study, all the dimensions of GTP were correlated with the hours of play per day. This finding shows the relevance of hours played in the manifestation of GTP in various dimensions. Interestingly, like neural adaptations that tend to be heightened as the period of exposure lengthens (Champney et al., 2007), the “outer intrusions/distortions” dimension, which includes phenomena such as seeing images with open eyes and perceptual distortions of physical environments, was associated with the time of exposure (i.e., session length). This finding may be explained by the neural adaptive mechanisms involved in this type of GTP experience (Ortiz de Gortari, 2017).

Convergent validity was supported by the fact that all the dimensions of GTP were significantly correlated with general INVWG, such as hallucinations, altered perceptions, imagery, and common sleep phenomena, as well as game-biased perceptions, such as associating physical places with game content or fantasizing about games. This finding suggests that those who are prone to general involuntary phenomena and biased perceptions are also susceptible to experiencing GTP.

### Impact of Game Transfer Phenomena

Finally, this study investigated the positive and negative impact of experiencing GTP and level of distress provoked by GTP. Most participants reported a positive impact of GTP, which coincides with previous studies that showed that players appraised GTP as a positive experience (Ortiz de Gortari and Griffiths, 2016), with some individuals even having tried to induce GTP for self-soothing experiences when restrained from gaming (Ortiz de Gortari and Basche, 2021). Contrary to what was expected, all the GTP dimensions, including inner intrusions were significantly correlated with negative impacts. All dimensions were also correlated with positive impact. However, both “outer intrusions” and “dissociations/mix-ups” were correlated with distress. These findings suggest that the positive or negative impact of GTP on some area of daily functioning (e.g., work, relationships) does not depend on whether the GTP manifest as “inner intrusions/misperceptions” or “outer intrusions/distortions.” However, the dimension in which GTP manifest is important for the distress caused by GTP. Previous studies have also reported that worry and distress are related to seeing images with open eyes, hearing sounds or voices coming from external sources or nowhere, and responding to GTP as if they were real (Ortiz de Gortari et al., 2011, 2016; Ortiz de Gortari and Griffiths, 2014a,c).

## Limitations

There are limitations to the present findings. The meticulous identification of GTP means that some of the items measured slightly similar phenomena, leading to multicollinearity. However, it is important to bear in mind that distinguishing involuntary phenomena, such as hallucinations, from other perceptual experiences is subjective and complex. The validation of instruments assessing general unusual sensory phenomena has not been based on a clear-cut distinction between hallucinations and other perceptual experiences (Mitchell et al., 2017).

In addition, the sample was self-selected, which may have resulted in selection bias and social desirability. While females now make up almost half of the gaming population, males are usually predominant in such studies, including this one. Future studies should test the GTPMS in a sample with a better gender distribution. This is particularly important because body perception is different between females and males, which could influence body-related GTP (Diekhoff et al., 2019).

## Conclusion and future research

The analyses performed in this study showed that the present GTP dimensions along the spectrum of imagery, misperceptions, perceptual distortions, hallucinations, and dissociations, which are based on a consideration of the perceived location and involvement of external stimuli as triggers, are reliable and valid for the assessment of GTP experiences. The findings show that phenomena manifesting as internal experiences are more common, while those manifesting as externalized intrusions are less common.

In terms of differences among the dimensions of GTP, prolonged exposure to a game was only associated with phenomena manifesting as outer or externalized or perceptual distortions, rather than inner phenomena or dissociations/mix-ups. Future studies should try to explain why only this dimension of GTP is associated with session length, and whether the neural adaptation mechanism plays a role.

Regarding the similarities between the dimensions of GTP, the findings revealed that all the dimensions were correlated with the susceptibility to experience INVWG and hours played per week.

The findings showed that independently of the dimension of GTP the impact can be positive or negative, although inner intrusions were not associated with distress. Future studies should take a closer look at the characteristics of GTP (e.g., duration, vividness such as volume and clarity), the role of the video game content and features, and the coping strategies players use to deal with GTP, to better understand whether GTP can be beneficial or problematic.

Furthermore, it is important to understand the continuum from episodic intrusions with game content (e.g., images, sounds, and impulses) to the engrossment in mental actions and imagery with game content that can lead to absentmindedness when engaging in activities that require full concentration and manual dexterity. It is also essential to understand the impact of intrusions with game content that can lead to sensations of unreality and impulsive actions in moments of confusion between the physical and the virtual world (Ortiz de Gortari and Griffiths, 2019).

This study showed that distress was associated with the “outer intrusions/distortions” and “dissociations/mix-ups” dimensions, which may be explained because players can easily interpret these two dimensions of GTP as not self-generated. These forms of GTP can also involve behaviors toward what players see or hear that lead to feelings of being out of control or ending up in embarrassing or risky situations (Ortiz de Gortari and Griffiths, 2019).

It is fundamental to understand the degree of which the assessment of GTP can support the identification of factors that contribute to the maintenance of gaming disorder (GD) symptoms and relapse. Various studies have shown associations between GD and GTP (Ortiz de Gortari et al., 2016; Jones-Rincon et al., 2021; Ortiz de Gortari and Gackenbach, 2021; Llamas-Alonso et al., 2022) and GTP have emerged as a mediator of factors for gaming disorder (e.g., gaming features and emotion regulation) (Cudo and Zabielska-Mendyk, 2022). For instances, the assessment of GTP in clinical cases of GD has shown to be beneficial for understanding the evolution of patient symptomatology and establishing differential diagnoses of psychosis (Ortiz de Gortari and Basche, 2021, 2022). Some forms of GTP resemble symptoms of addiction. For example, players display selective attention toward game-related cues suggesting cue reactivity in everyday life, and visual and auditory imagery with game content appear to highlight processes of cravings. Moreover, certain forms of GTP resemble sensory disturbances as side effects of hallucinogens (hallucinogen persistent perceptual disorder) and alcohol withdrawal.

## Data availability statement

The raw data supporting the conclusions of this article will be made available by the authors, without undue reservation.

## Ethics statement

The studies involving human participants were reviewed and approved by the Ethics Committee of the Faculty of Psychology, Speech Therapy and Education Sciences of the University of Liège. The patients/participants provided their written informed consent to participate in this study.

## Author contributions

AO: conceptualization, methodology, item creation, data collection, original draft composition, and writing. ÅD: statistical analysis, text editing, and reviewing. Both authors contributed to the article and approved the submitted version.
